# Capacity optimization strategy for gravity energy storage stations considering the impact of new power systems

**DOI:** 10.1371/journal.pone.0320734

**Published:** 2025-04-23

**Authors:** Can Lv, Jun He, Jingjing Ma, Yukun Yang, Fan Liu, Wentao Huang

**Affiliations:** Hubei Collaborative Innovation Center for High-Efficiency Utilization of Solar Energy, Hubei University of Technology, Wuhan, China; Hanshan Normal University, CHINA

## Abstract

The integration of renewable energy sources, such as wind and solar power, into the grid is essential for achieving carbon peaking and neutrality goals. However, the inherent variability and unpredictability of these energy sources pose significant challenges to power system stability. Advanced energy storage systems (ESS) are critical for mitigating these challenges, with gravity energy storage systems (GESS) emerging as a promising solution due to their scalability, economic viability, and environmental benefits. This paper proposes a multi-objective economic capacity optimization model for GESS within a novel power system framework, considering the impacts on power network stability, environmental factors, and economic performance. The model is solved using an enhanced Grasshopper Optimization Algorithm (W-GOA) incorporating a whale spiral motion strategy to improve convergence and solution accuracy. Simulations on the IEEE 30-node system demonstrate that GESS reduces peak-to-valley load differences by 36.1% and curtailment rates by 42.3% (wind) and 18.7% (PV), with a 15% lower levelized cost than CAES. The results indicate that GESS effectively mitigates peak load pressures, stabilizes the grid, and provides a cost-effective solution for integrating high shares of renewable energy. This study highlights the potential of GESS as a key component in future low-carbon power systems, offering both technical and economic advantages over traditional energy storage technologies.

## 1. Introduction

In recent years, industrialization, urbanization and population growth are taking place worldwide. These increases have a direct impact on ecosystems and the human condition, as the demand for energy, mainly fossil, is becoming greater, resulting in a significant increase in installed wind and solar power capacity globally [1]. Wind and solar power can effectively ease dependence on fossil fuels and reduce CO2 emissions from fossil fuels.

However, the increased penetration of intermittent renewable energy sources such as solar and wind in the distribution system (DC) and the high variability of intermittent loads can lead to various problems such as increase in power slope, voltage fluctuations, reverse current, etc [2]. In order to effectively overcome these problems, energy storage systems (ESS) have been widely incorporated into new power system designs [3]. In addition, ESS helps manage power fluctuations associated with the addition of RES to the grid, mitigates Duck-curve mitigation, shifts power from surplus to deficit periods, aids in voltage regulation, peak shaving, demand-side response, deficit reduction, and aids in frequency regulation for new power systems [4]. In ESS gravity energy storage systems (GESS) are more advantageous in terms of siting, scale and economics compared to battery energy storage systems (BESS) and compressed air energy storage (CAES) [5]. In different ESS, pumped storage (PHS) and CAES have been practically used and developed [6]. 99% of the world’s total energy storage capacity is of the PHS type, with other forms of energy storage accounting for 1%. Studies have shown that GESS can also be used as an alternative system to PHSS as it is not only free from regional constraints and adverse environmental impacts, but also the technology has a long service life, high efficiency and fast sub-second response, low cost of storage (LCOS), low O&M costs, and no pollutant emissions. The different types of GES are hydraulic GES, lifting GES, deep sea GES, underground GES and orbital GES, but most of them are in their infancy [4,7–9].

In 24 years, the share of renewable energy in China’s total energy consumption accounted for 26.4%, up 0.4 percentage points compared with last year. New energy sources used for grid-connected power generation accounted for 47.62% of the total energy generated. The installed capacity of new energy generation has been growing steadily since China began implementing its “peak carbon and carbon neutral” policy [3]. In the face of continued growth, the search for more cost-effective energy storage systems will enhance the ability of new energy sources to absorb grid shocks and further accelerate the development of new energy power plants around the world. E.N. Nyeche examines a system in Nigeria that integrates photovoltaics, wind turbines, and a PHS to provide stable power at a LCOS of $0.27/kWh [10]. Tobias Massier replaces stationary energy storage systems with electric vehicle energy storage plants and provides insights into battery aging in electric vehicles to increase the penetration of new energy generation [11]. Kwon Ochang is an integrated system that uses a micro gas turbine (MGT) and an energy storage system (ESS) that reuses batteries to reduce duck curves in the power system and stabilize the power system so that it can generate power flexibly [12]. Sadeghi S & Askari B I studied an integrated system based on PV-CAES coupled molten carbonate fuel cell-gas turbine and battery [13]. Studies have shown that the required power should be released from the fuel cell at the beginning of the discharge process and then the CAES can be engaged to supply power.

Although various aspects of PHS and CAES system integration have been studied in various ways, research on GESS is still in its infancy. This type of energy storage method is mainly categorized as dry and wet. Wet GESS include hydraulic, deep-sea and suspended, while dry GESS are underground, rail, tower and rack [14]. Berrada A et al. investigated the dynamic modeling of GESS when integrated with a PV plant.The Matlab/Simulink program was used to evaluate the performance of the hybrid system including the PV plant, the GES system and the grid [15]. Emrani et al. studied and analyzed the performance of wire rope hoisting GES under dynamic conditions. They evaluated the performance of GES systems with and without wire rope hoisting. The results show that the energy capacity of the GES with wire rope lifting is twice that of the GES without wire rope lifting [16]. Mixed renewable energy systems and GES system optimization are then presented in the study [17]. which compares the optimal design of GES and fuel cells, they minimized GES construction costs by applying mechanical loads to the system structure [18]. They also developed a new methodology to optimize GES systems integrated with photovoltaic wind farms and developed a system for optimizing GES systems integrated with photovoltaic wind farms [19]. They investigated that the levelized storage cost of GES varies between 7.5 €ct/kWh and 15 €ct/kW, while gravity storage using a wire suspension system (GESH) varies between 3.8 €ct/kWh and 7.3 €ct/kWh. The LCOS of GES and GESH were then compared with other energy storage systems. The results obtained show that GESH is cost competitive with pumped storage and compressed air energy storage technologies. The analysis performed also shows that both GES and GESH offer interesting economic advantages for the provision of energy arbitrage services [20].

Among the existing common algorithms, the Adaptive Lightning Attachment Procedure Optimizer has an intuitive model, but its search mechanism is singular. The hybrid moth - flame algorithm with particle swarm optimization combines advantages and has good search capabilities, yet it faces difficulties in parameter adjustment. The Grey Wolf Optimization Algorithm has an intuitive concept and strong exploration ability in the early stage, but it has large convergence fluctuations and is prone to being trapped in local optima. The Chaotic Chimp Sine Cosine Algorithm has unique advantages in balancing global and local search, however, its search strategy is relatively fixed [21–28]. In contrast, the Grasshopper Optimization Algorithm (GOA) has remarkable advantages. GOA simulates the behavior of grasshopper swarms. Its search mechanism is diverse and can break through the limitations of a single - mode, comprehensively capture key features in complex power problems, widely explore the solution space, and effectively avoid the problem of limited search scope. In terms of parameter adjustment, GOA has few parameters and low sensitivity. It does not require a large number of attempts, can converge stably, and reduces the application difficulty and cost.

Despite these advancements, critical gaps remain: Firstly, existing studies predominantly focus on wet GESS, while dry GESS remain underexplored, despite their broader geographic applicability.Secondly, most optimization models for GESS prioritize single objectives, neglecting the multi-objective trade-offs between economic, environmental, and grid-impact factors.Lastly,traditional algorithms struggle with the nonlinear constraints and high-dimensional search spaces inherent in GESS capacity optimization, often leading to suboptimal solutions. This study addresses these gaps by proposing a novel multi-objective optimization framework for tower-based GESS capacity allocation in hybrid wind-PV-thermal power systems. We develop a hybrid grid-connected model that incorporates tower-based GESS to enhance renewable energy absorption and grid stability, filling the gap in dry GESS applications. A holistic objective function is formulated, balancing power network impacts, environmental costs, and economic factors. By integrating the whale spiral motion strategy into GOA, we overcome the limitations of traditional algorithms in handling complex constraints, achieving faster convergence and higher accuracy.

In general, the analysis of the ability and benefits of new energy consumption by installing mechanical energy storage technologies, including GESS, on the generation side of new energy systems has hardly been addressed in the literature, which is undoubtedly a big gap. In addition, the literature in this paper pays less attention to dry GESS for wet GESS, which makes there is a gap in the research on the performance of this type of GESS in terms of technical and economic benefits when it is coupled with the generation side of new energy systems. Therefore, the originality of this work lies in the introduction of a new type of power system generation side and GESS environmentally integrated system, which not only produces green and reliable power for the grid at the highest efficiency, but also minimizes the volatility penalties and instability associated with new energy sources connected to the grid, resulting in the highest returns and a stable power market. Of all the dry GESS types, the hanging tower GESS was chosen for its suitability for almost all parts of the globe.

## 2. Considering a multi-objective optimization model for GESS under a novel power system

### 2.1. The GES system under study

**Fig 1:**
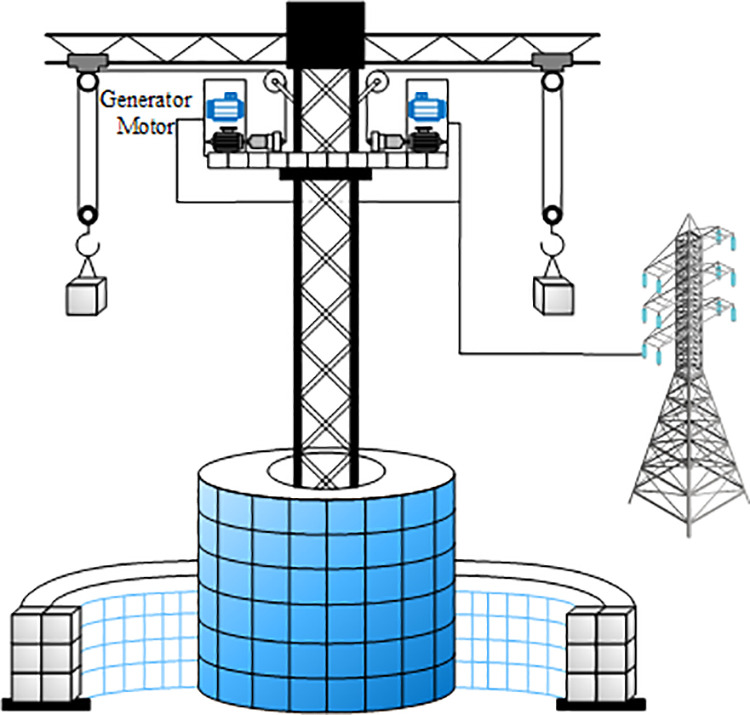
GESS diagram of the study.

The studied GES system consists of six cranes, a large number of concrete blocks, and several motors. An overview of the studied GES system is further shown concisely in Fig 1. One of the center towers is the blue part, which we’ll call Tower A, made of giant piles of discarded concrete blocks. In gray is the stacking tower for bricks from tower a, which we call tower b. The tower is a hollow cylinder. The core working principle is the use of a crane to lift concrete blocks from the lower hollow cylindrical tower and store them in a central tower for energy storage. When energy is required, the crane releases the bricks from the central tower to the lower tower, where the potential energy of the bricks is converted into electrical energy by means of a motor/generator. This process is precisely controlled by specialized control software that ensures that the bricks are placed in exactly the right place every time to keep the system running efficiently. The motors and generators are located on high platforms and are connected to the new energy power plant and the grid. Through this connection, the GES system is able to store redundant power from new energy plants, regulate the impact of new energy sources on the utility grid, and participate in regulation services in the electricity market. The GES system not only enhances the efficiency of energy utilization, but also provides a stable power supply when power demand fluctuates. Its application is promising and not only provides strong support for the large-scale application of renewable energy, but also guarantees the stability and reliability of the power system.

**Fig 2:**
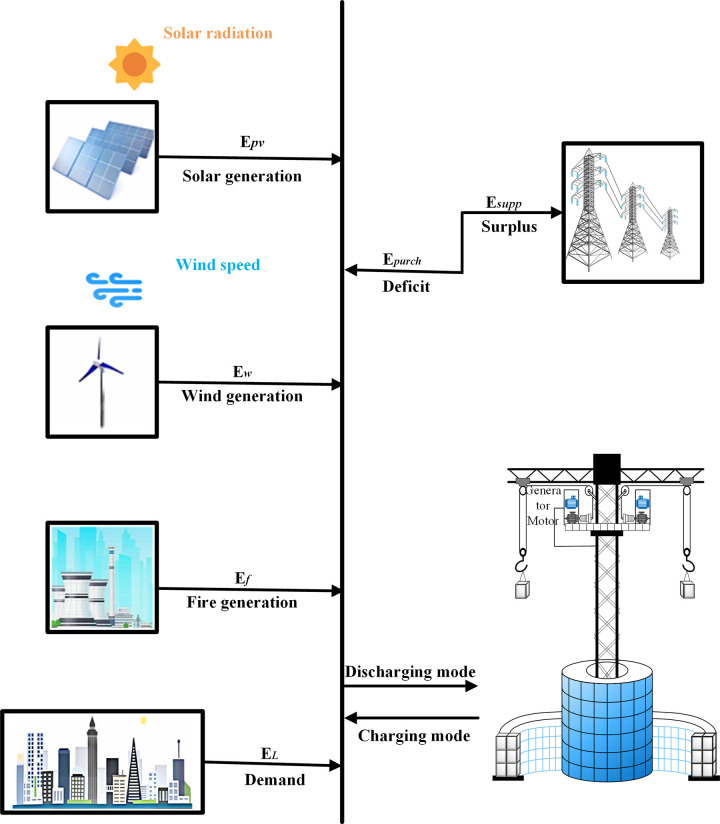
Schematic diagram of GESS coupled with wind-fire hybrid power plant.

Finally, the GESS is integrated into a hybrid grid-linked system consisting of thermal units, wind turbines (WTs), photovoltaic modules (PVs), and defined load demands. Fig 2 shows the schematic and power flow of this hybrid integrated system. Energy is generated and then rationed to allocate feed-in power according to the demand load at the time, with any excess power being stored by the GES and fed into the grid during peak demand periods and at the peak of interest, improving both system balance and reliability, and plant revenues.

### 2.2. Objective function of GESS

**Fig 3:**
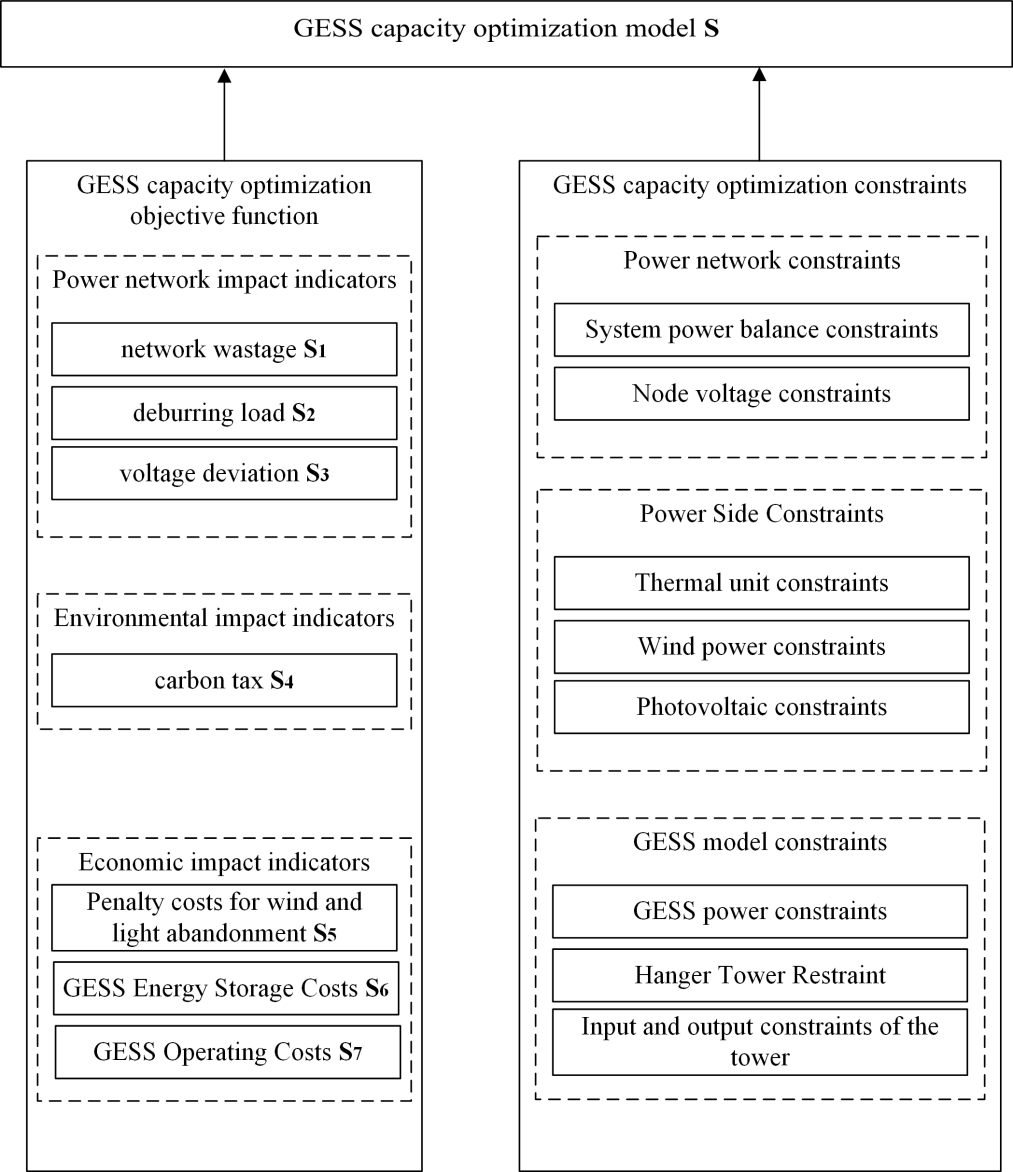
Block diagram of GESS capacity optimization model.

GESS distribution optimization strategy is to achieve under certain load conditions, through the regulating role of the GES system, considering the supply-side benefit maximization and the consumption of new energy to balance the fluctuation of the grid, integrated power network impact indicators, environmental impact indicators, economic impact indicators and other multi-directional impacts, to construct the GESS optimization model, the specific optimization model is shown in Fig 3.

In practical capacity optimization, the objective function is optimized to a minimum as follows [29]:


$$\{\begin{array}{l} S=\min(k_{1}S_{1}+k_{2}S_{2}+k_{3}S_{3}+k_{4}S_{4}+k_{5}S_{5}+k_{6}S_{6}+k_{7}S_{7})\\ k_{1}+k_{2}+k_{3}+k_{4}+k_{5}+k_{6}+k_{7}=1 \end{array}$$ (1)

In the Formula, k_1_, k_2_, ……, k_7_ are the weighting factor for each of the above indicators.

In GESS capacity optimization, Analytic hierarchy process (AHP) is used to calculate the weight coefficients of each impact indicator


$$k_{z}=\frac{\sqrt[{Z}]{\prod_{y=1}^{Z}a_{zy}}}{\sum_{z=1}^{Z}\sqrt[{Z}]{\prod_{y=1}^{Z}a_{zy}}},z=1,2,\cdots ,Z$$ (2)

In the Formula, k_z_ is the weight coefficient of the z weighted impact indicator; $a_{zy}$ is the value of the importance result comparison between element z and element y within the AHP judgment matrix; Z is the matrix order.

At the power network level: S_1_ is the system network loss, which is an important economic objective at the grid level to reduce power losses and resource wastage; S_2_ is the load shedding, which is a contingency measure in the power system to maintain system stability and avoid large-scale power outages. When the system is overloaded, under-generated, or in the event of other emergencies, the supply and demand are balanced by actively reducing or disconnecting portions of the load, preventing the power system from collapsing. S_3_ is the voltage deviation, and S_3_ is mainly used to ensure that the voltage level at each node in the power system is kept within a reasonable range, thus improving the operational stability and reliability of the power system.

At the level of environmental impact: set S_4_ as the smallest carbon tax, there are many international issues that only consider the smallest carbon emissions and ignore the supply-side benefits, and there are few domestic and foreign literatures on the optimization of gravity energy storage capacity that consider the carbon tax issue, S_4_. Its purpose is to minimize the total amount of carbon tax while meeting certain environmental protection requirements and economic benefits, mainly standing on the supply-side benefits and overall environmental protection considerations, and is a fundamental condition for the program’s high environmental protection and high benefits.

At the economic impact level: let S_5_ be the penalty cost of wind and light abandonment; S_6_ be the cost of GESS energy storage; S_7_ be the cost of GESS operation, and enhance the economic benefits brought by gravity energy storage as much as possible through the capacity optimization strategy.

#### 2.2.1. Power network impact indicators.

(1) Net loss S_1_:


$$S_{1}=\sum_{i\in N_{L}}G_{ij}\left(U_{i}^{2}+U_{j}^{2}-2U_{i}U_{j}\cos\left(\theta_{i}-\theta_{j}\right)\right)$$ (3)

where $U_{i},\;U_{j}$ are the voltage amplitude of nodes i and nodes j, respectively; $G_{ij}$ is the conductance of branch circuits between nodes i, j; $N_{L}$ is the set of transmission lines; $\theta_{i},\;\theta_{j}$ are the voltage phase angle of nodes i, j.

(2) Chipping load S_2_:


$$S_{2}=\sum_{t=1}^{T}(P_{p,t}\cdot \eta_{p}+P_{v,t}\cdot \eta_{v}�$$ (4)

Where T denotes the total number of optimization time periods, $P_{p,t},\;P_{v,t}$ denote the peak load and valley load at the moment of t, $\eta_{p},\;\eta_{v}$ denote the peak load weighting coefficient and valley load weighting coefficient at the moment of t, respectively.

(3) Voltage deviation S_3_: It is mainly used to ensure that the voltage level of each node in the power system is maintained within a reasonable range, thus improving the operational stability and reliability of the power system. It is specifically expressed in the following form:


$$\{\begin{array}{l} S_{3}=\frac{S_{a1}}{NT}\sum_{i=1}^{N}\sum_{t=1}^{T}\frac{\left(U_{t,i}-U_{t,i\min})(U_{t,i\max}-U_{t,i}\right)}{\left(U_{N}-U_{t,i\min})(U_{t,i\max}-U_{N}\right)}\\ S_{a1}=\frac{1}{NT}\sum_{i=1}^{N}\sum_{t=1}^{T}\left|\frac{U_{t,i}}{U_{N}}-\bar{U_{i}}\right|\\ \bar{U_{i}}=\frac{1}{T}\sum_{t=1}^{T}\left|\frac{U_{N}-U_{t,i}}{U_{N}}\right| \end{array}$$ (5)

where N is the number of nodes in the distribution network; U_t,i_ is the voltage deviation of node i at moment t; U_t,i min,_ U_t,i max_ are the minimum and maximum values of the rated voltage deviation of node i at moment t, respectively; U_N_ is the rated voltage; $S_{a1}$ is the voltage fluctuation level index; $\bar{U_{i}}$ is the average value of the voltage fluctuation level of node i in the calculation time period.

#### 2.2.2. Environmental impact indicators.

In response to climate change, many countries and regions have implemented carbon tax policies to reduce greenhouse gas emissions by taxing carbon dioxide emissions. Setting a carbon tax minimum objective function can effectively help formulate a reasonable carbon tax policy to achieve the emission reduction target while minimizing the negative impact on the economy.

The carbon tax minimizes the objective function of S_4_:


$$S_{4}=C_{tax}E_{i}\left(P_{fi}\right)$$ (6)

$$E_{i}\left(P_{fi}\right)=aP_{fi}^{2}+bP_{fi}+c$$ (7)

Where: a, b, c are carbon emission factors, $E_{i}(P_{fi})$ is the carbon emission of thermal power units in a given time period, $C_{tax}$ is the market price of carbon tax.

#### 2.2.3. Economic impact indicators.

(1) Wind and light abandonment penalty costs S_5_: The amount of abandoned wind and light is an important indicator to measure the new energy power generation system new energy consumption ability, the smaller the amount of abandoned light, the greater the ability to consume new energy, abandoned wind and light penalty coefficient is also known as the wind power photovoltaic priority coefficient, due to the limited level of loads in the regional power grid, adding abandoned wind and light penalty coefficient in the objective function can ensure that the wind power photovoltaic priority consumption, effectively limiting the amount of wind and light abandonment of the system, and its physical meaning is the penalty when the power system abandons a unit of wind power. Therefore when this coefficient increases, the more effective the consumption of new energy in the system.


$$S_{5}=F_{pv}\cdot \sum_{d=1}^{D}\sum_{t=1}^{n}P_{pv-q}(t)+F_{\text{w}}\cdot \sum_{d=1}^{D}\sum_{t=1}^{n}P_{w-q}(t)$$ (8)

Where: $F_{pv}$ is the penalty coefficient of abandoned light, $P_{pv-q}(t)$ is the abandoned light power in time period t, $F_{w}$ is the penalty coefficient of abandoned wind.

(2) GESS energy storage cost S_6_: The energy storage power and energy storage tariff of the GES system are closely related to the cost of energy storage:


$$S_{6}=\sum_{t=1}^{n}\left|P_{pt}\right|C_{gt}\Delta t$$ (9)

where $C_{gi}$ is the energy storage tariff of the gravity energy storage system at time t, $C_{gt}=0.25C_{t}$, $C_{t}$ is the time-sharing tariff at time t, $P_{pt}$ is the energy storage power of the gravity energy storage system at time t.

(3) GESS Running Costs S_7_:


$$S_{7}=F_{P}\left(t\right)P_{p-power}$$ (10)

$$F_{P}\left(t\right)=C_{lift}\left(t\right)+C_{release}\left(t\right)$$ (11)

Where $F_{p}\left(t\right)$ is the operating cost of the GES system at time t, $P_{p-power}$ is the installed capacity of the GES system. $C_{lift}\left(t\right)$ is the cost of weight lifting at time t, $C_{release}\left(t\right)$ is the cost of weight releasing at time t.

### 2.3. Constraints of the GESS optimization model

#### 2.3.1. Power network constraints.

(1) System power balance constraints [30]:


$$P_{h}(t)+P_{w}(t)+P_{pv}(t)+P_{f}(t)=P_{LD}(t)+P_{p}(t)+P_{loss}(t)$$ (12)

Where: $P_{h}(t)$ is the output power of gravity energy storage, $P_{w}(t)$ is the output power of wind, $P_{f}(t)$ is the output power of thermal power plant, $P_{pv}(t)$ is the output power of photovoltaic, $P_{LD}(t)$ is the load of new power system, $P_{P}(t)$ is the power consumed by gravity energy storage. $P_{loss}(t)$ represents the network loss in the system.

(2) Node voltage constraint:


$$U_{t,i\min}⩽U_{t,i}⩽U_{t,i\max}$$ (13)

where $U_{t,i\min}$ is the minimum value of i node voltage at time t, $U_{t,i}$ is the i node voltage at time t, $U_{t,i\max}$ is the maximum voltage of the i node at time t.

#### 2.3.2. Power side constraints [30].

(1) Thermal power unit constraints:


$$P_{fi,\min}\leq P_{fi}\left(t\right)\leq P_{fi,\max}$$ (14)

Where $P_{fi,\max}$_,_ $P_{fi,\min}$ are the maximum and minimum outputs of the first thermal power unit.


$$P_{f}(t+1)-P_{f}(t)\leq \Delta P_{f,\text{up}}\Delta t$$ (15)

$$P_{f}(t)-P_{f}(t+1)\leq \Delta P_{f,\text{down}}\Delta t$$ (16)

Where $\Delta P_{f,\text{up}}$ is the upward creep rate of the thermal unit, $\Delta P_{f,\text{down}}$ is the downward creep rate of the thermal unit.

(2) Wind power constraints:


$$0\leq P_{w}(t)\leq P_{w-power}$$ (17)

Where $P_{w-power}$ is the maximum power of the wind turbine.

(3) Photovoltaic constraints:


$$0\leq P_{pv}(t)\leq P_{pv-power}$$ (18)

Where $P_{pv-power}$ is the maximum power of the photovoltaic unit.

#### 2.3.3. GESS model constraints.

(1) GESS power constraints:


$$u_{h,t}P_{h}^{\min}\leq P_{h,t}\leq u_{h,t}P_{h}^{\max}$$ (19)

$$u_{\text{p},t}P_{\text{p}}^{\min}\leq P_{\text{p},t}\leq u_{\text{p},t}P_{\text{p}}^{\max}$$ (20)

Where $P_{h}^{\min},P_{h}^{\max}$ are the maximum and minimum generation power allowed for gravity storage, $P_{\text{p}}^{\min},P_{\text{p}}^{\max}$ are the maximum and minimum lifting power allowed for gravity storage. $u_{h,t}$ denotes whether or not the gravity storage is generating power at time period t, $u_{\text{p},t}$ denotes whether or not the gravity storage is storing power at time period t.

(2) Lifting tower capacity constraints:


$$r_{\min}⩽r_{t}⩽r_{\max}$$ (21)

Where r is the tower capacity at time t, and $r_{\min},r_{\max}$ are the upper and lower limits of tower capacity.

(3) Lifting tower input and output constraints: Gravity storage units cannot simultaneously lift and generate electricity and need to be constrained


$$u_{h,t}+u_{\text{p},t}\leq 1$$ (22)

where $u_{h,t}$ denotes whether the gravity storage is generating electricity at time period t and $u_{\text{p},t}$ denotes whether the gravity storage is storing energy at time period t.

## 3. Methods

### 3.1. Grasshopper optimization algorithm

The capacity allocation problem in power systems usually involves the allocation of different power devices. These devices need to be configured according to their respective requirements and constraints to meet the requirements and optimization goals of the power system. From the above equation, it is known that the required objective function type nonlinear planning model, the decision variables include thermal, wind, photovoltaic and GESS capacity, and the constraints are complex. Thus the model solution belongs to the generalized assignment problem (GAP). Since GAP is very complex and it is generally difficult to find the global optimal solution, approximation algorithms or origin meta-heuristic optimization methods are tried to find more solutions [31]. Considering the scale degree, complex constraints and solution requirements of the GESS capacity allocation problem, this paper selects the grasshopper algorithm in meta-quick optimization for improvement and solves the system model.

GOA is a meta-heuristic bionic optimization algorithm proposed by Prof. Mirjalili in 2017, which focuses on the simulation of grasshopper locomotor behavior in nature [32]. In nature, the movement range of juvenile grasshoppers is small, while the movement range of adult grasshoppers is relatively large, based on which the algorithm maps the movement behavior of juvenile grasshoppers to local search and the movement behavior of adult grasshoppers to global search.

In the field of power system optimization, although the Genetic Algorithm (GA), PSO, and Differential Evolution (DE) have their respective applications, when compared with the GOA, the advantages of GOA are obvious. When dealing with the distribution network reconfiguration problem with distributed generation and complex loads, due to the relatively fixed genetic operations of GA, some high - quality combination schemes of distributed generation access are likely to be overlooked in the initial search stage. This makes it difficult for subsequent evolution to jump out of the local optimal solution, and it is impossible to achieve the optimal reconfiguration of the distribution network to reduce power losses. PSO is greatly affected by the initial positions and velocities of particles. In a certain urban power grid planning case, due to the premature aggregation of particles, the planned locations of substations and the layout of transmission lines are unreasonable, and the future load growth demand cannot be met. When facing the high - dimensional and complex economic dispatch problem of the power system, the convergence performance of DE drops sharply, and it is difficult to find the global optimal solution with the lowest cost and the highest benefit among numerous unit combination and power output allocation schemes.

In contrast, GOA performs outstandingly in the same complex power system scenarios. Its search mechanism, which simulates the behavior of grasshopper swarms, can explore different access points and capacity configurations from multiple perspectives when dealing with the access optimization of wind farms and photovoltaic power stations, and find the best new energy access solutions, thereby enhancing the clean energy absorption capacity of the power system. In terms of convergence performance, when solving the hydro - thermal power joint dispatching problem, GOA can converge stably to the global optimal solution, and reasonably arrange the generation ratio of hydro - power and thermal - power, which not only meets the power demand but also reduces the power generation cost. Moreover, GOA has few parameters and low sensitivity. In the operation optimization of power systems with different seasons and different load characteristics, it can operate stably without repeated parameter adjustments, which significantly reduces the algorithm application difficulty and cost, and provides a more efficient and reliable solution for power system optimization.

This algorithm has the property of being simple, uncomplicated and easy to implement, which allows for a great deal of flexibility when it comes to improving it for specific problems.The GOA position update expression is as follows:


$$Y_{i}^{d}=c\left(\sum_{j=1}^{N}c\frac{ub_{d}-lb_{d}}{2}s\left(\left|y_{j}^{d}-y_{i}^{d}\right|\right)\frac{y_{j}-y_{i}}{d_{ij}}\right)+{\hat{T}}_{d}$$ (23)

$$c=c_{\max}-l\frac{c_{\max}-c_{\min}}{L_{\max}}$$ (24)

$$s(r)=fe^{-\eta \hbar }-e^{-r}$$ (25)

Where is the contraction coefficient; ub_d_ and lb_d_ are the upper and lower limits of the grasshopper’s locomotion behavior; s(r) is the interaction force function between different individuals, where F and H are the attraction strength and attraction scale; d_ij_ is the distance between different grasshoppers; ${\hat{T}}_{d}$ is the optimal solution in the D-dimensional space in the current state; l is the number of iterations, and L_max_ is the maximum number of iterations.

### 3.2. Improvements in GOA

Since GOA was proposed, numerous scholars have studied it in depth and confirmed its effectiveness in solving constrained optimization problems and its excellent performance in global search. However, the algorithm still has some shortcomings. Its search efficiency is not high in the local optimization process and it is easy to fall into the local optimum. Xu Zhangze et al. had proposed a grasshopper optimization algorithm based on spiral motion, which improves the local search capability of this algorithm, but the literature is only a one-dimensional spiral and does not consider the whole search space [33]. On this basis, the whale spiral trajectory is added to extend the optimization process to the full dimension, which enhances the global optimization efficiency of the algorithm and takes into account the completeness of the optimization to prevent falling into the local optimum, and the position is updated in the following way:


$$X(t+1)= \{\begin{array}{c} X_{q}(t)-A\cdot D\left[p<0.5\right]\\ D^{'}\cdot e^{bl}\cdot \cos(2\pi l)+X_{q}(t)\left[p\geq 0.5\right] \end{array}$$ (26)

$$X(t+1)=X_{rand}(t)-A\cdot D$$ (27)

$$D=\left|C\cdot X_{rand}(t)-X(t)\right|$$ (28)

where X_rand_ (t) denotes the installed capacity of gravity energy storage from the current moment.

When t < T, automatically update parameters A, C, l, and p. When p < 0.5, if A < 1, update the locust’s position based on the ‘searching for prey phase’; if A ≥ 1, update the locust’s position based on the ‘spiral hunting phase.’ The locust’s action is determined by p, allowing for synchronized updates of contraction encirclement and spiral hunting. When p ≥ 0.5, update the individual locust’s position according to the ‘encircling prey phase,’ calculate the current total cost, and record the current locust position corresponding to the optimal gravity energy storage capacity under the current scheme. If t < T, an optimal capacity is obtained; if t > T, set t = t + 1 and restart the calculation.

**Fig 4:**
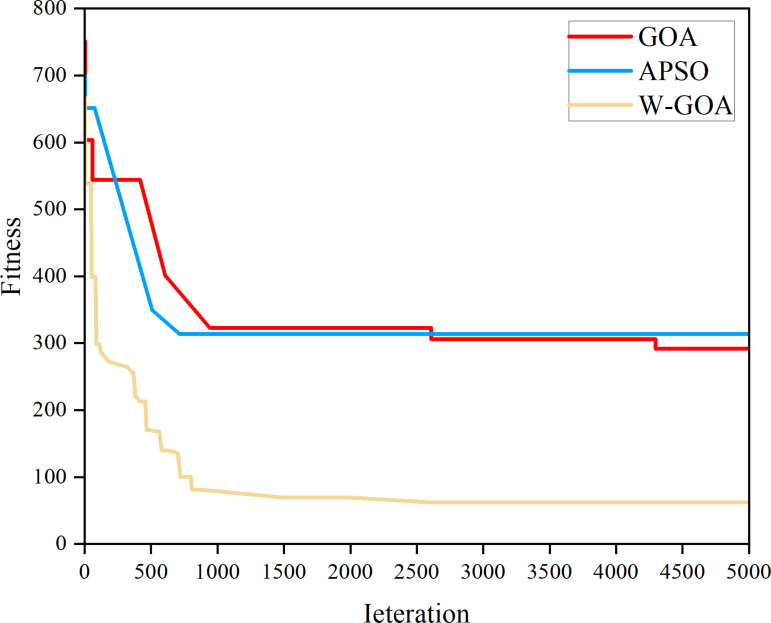
W-GOA, GOA, PSO iteration curves.

The Whale-Grasshopper Optimization Algorithm (W-GOA) proposed in this paper combining the Whale Optimization Algorithm Spiral Motion Strategy is compared with the traditional GOA and Adaptive Particle Swarm Optimization PSO, and three optimization algorithms are used to solve the test function respectively, and the optimization results are shown in Fig 4. The W-GOA algorithm can continuously outstrip of the local traps and iterate towards this global optimum, and the convergence speed and solution are also faster.

**Fig 5:**
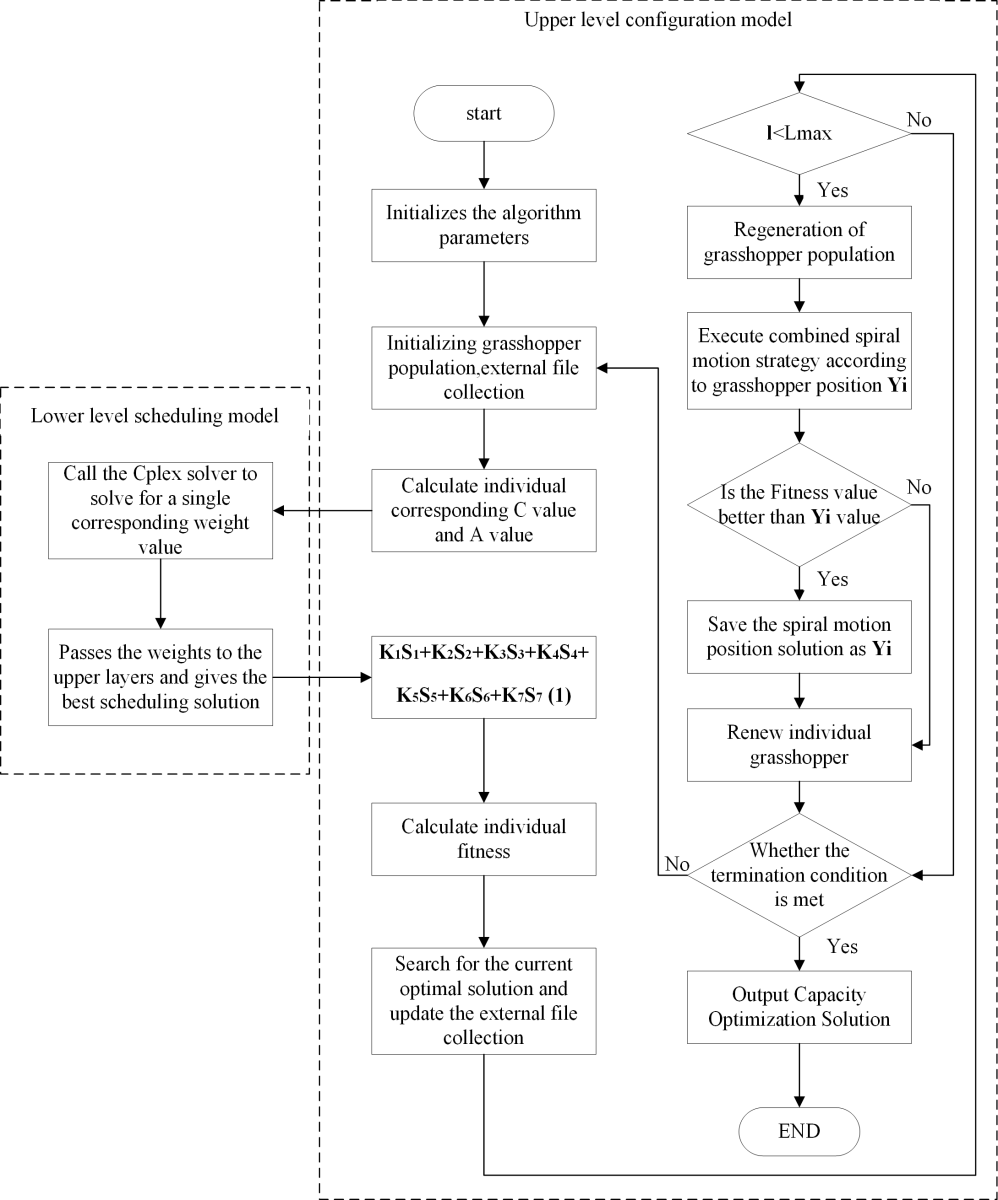
W-GOA flow chart.

W-GOA is used for this capacity model optimization is divided into two layers of model and the flowchart of the allocation scheme is shown in Fig 5:

### 3.3. Specific steps

Step 1: First initialize the algorithm parameters. Set the coefficients; the maximum number of iterations, the strength of attraction and the ratio are set to and respectively; the helix radius, the helix angle;

Step 2: Initialize the grasshopper quantity to 30, set the initial data and update the external fileset. The unit configuration capacity is compared to the grasshopper location in GOA. The dimension of the search D-space is determined by p. When p < 0.5, the position of the whale is updated according to the “prey search phase” when A < 1, and the position of the whale is updated according to the “spiral hunting phase” when A ≥ 1. When p ≥  0.5, the current total cost is calculated by updating the position of individual whales according to the “encircling prey phase”, and the current position of whales is recorded, which corresponds to the optimal installed capacity of gravity energy storage under the current scenario.

Step 3: Calculate with, and input the data into the lower scheduling model;

Step 4: The lower level model uses AHP to calculate the weight values of each parameter to obtain the weight coefficients at this point and return them to the upper level configuration model;

Step 5: The upper model calculates individual fitness values based on Equation S and updates the external file dataset;

Step 6: Determine if the maximum number of iterations has been reached. If yes, proceed to the next step; otherwise, skip to step 11;

Step 7: Update the grasshopper population to perform whale spiral motion control based on the current gasshopper position according to Eqs. 26–27;

Step 8: Determine if the current fitness value is better than the last position, if yes, proceed to the next step; otherwise go to step 9;

Step 9: Store the solution of the current helical motion position into Y_i_;

Step 10: Update the grasshopper individual;

Step 11: Determine if the termination conditions are met. If so, proceed to the next step; otherwise, return to step 2;

Step 12: Return the location of the best grasshopper individual and output the best solution.

## 4. Simulation arithmetic

### 4.1. Introduction to the arithmetic

**Fig 6:**
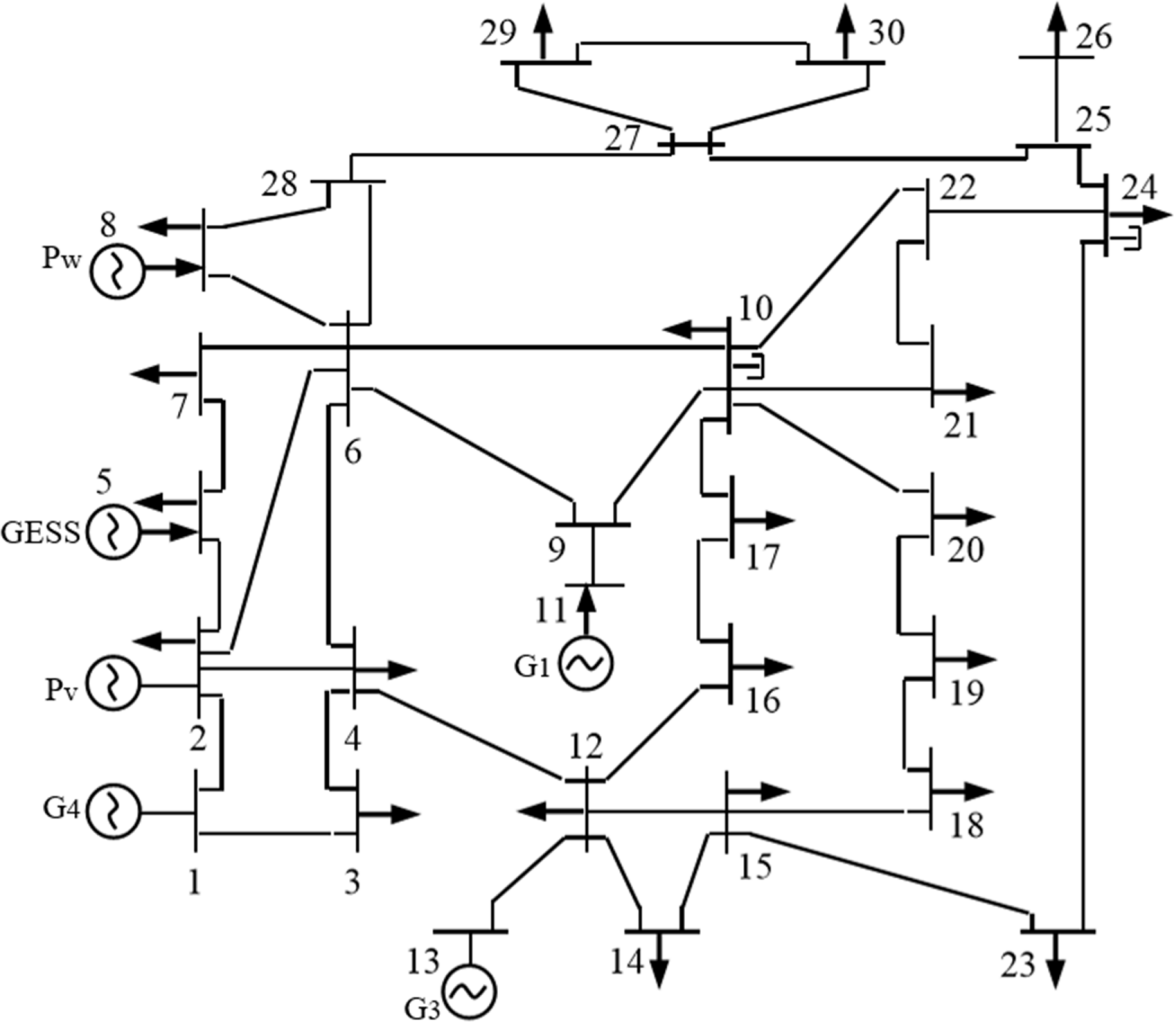
IEEE-30 node simulation system diagram.

The IEEE30 node system is used as an example to simulate and validate the gravity energy storage system model under the new power system, and the system is shown in Fig 6; based on the analysis of the improvement example of a power plant in Hubei Province.

**Fig 7:**
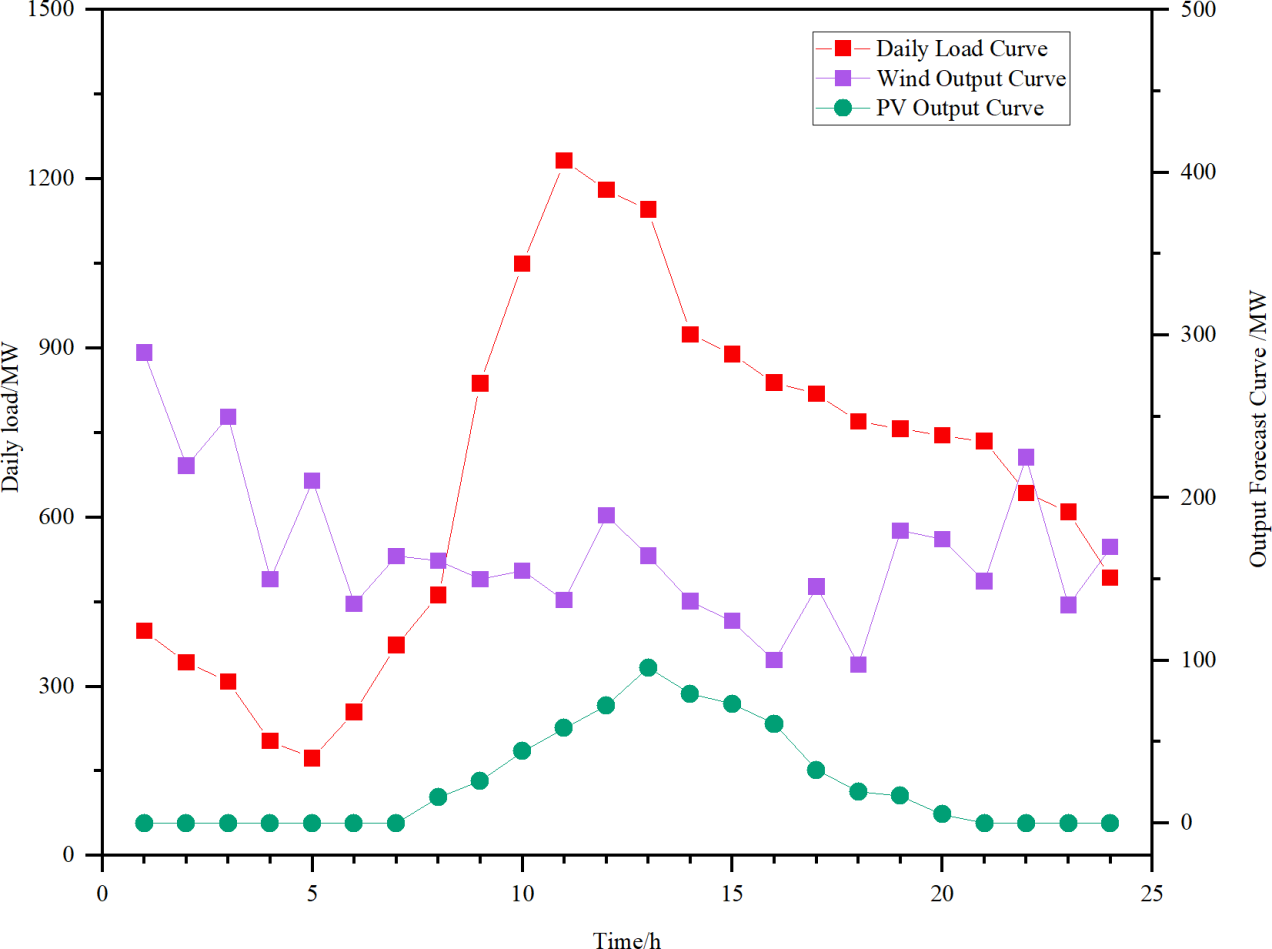
Daily load vs. wind PV output curve.

The system load forecast demand curve, wind power PV output forecast curve are shown in Fig 7. According to the load data of a region, the daily load curve is obtained with 24 moment points throughout the day to analyze the trend of the load and the time points of various load distributions, which can be used to plan the capacity and time points of gravity energy storage to participate in peaking based on the curve.

Load peaks between 10:00 and 22:00, and is lower and smoother between 23:00 and 09:00. Photovoltaic output will be affected by sunlight, power generation is concentrated in the daytime, and the output reaches the maximum at noon during the day, and the curve is relatively smooth, the distribution of output is more regular, and it has positive peaking characteristics. Wind power generation is more volatile, with high wind speeds in the evening, generating more power than at midday, with anti-peaking characteristics.

### 4.2. Analysis of results

**Fig 8:**
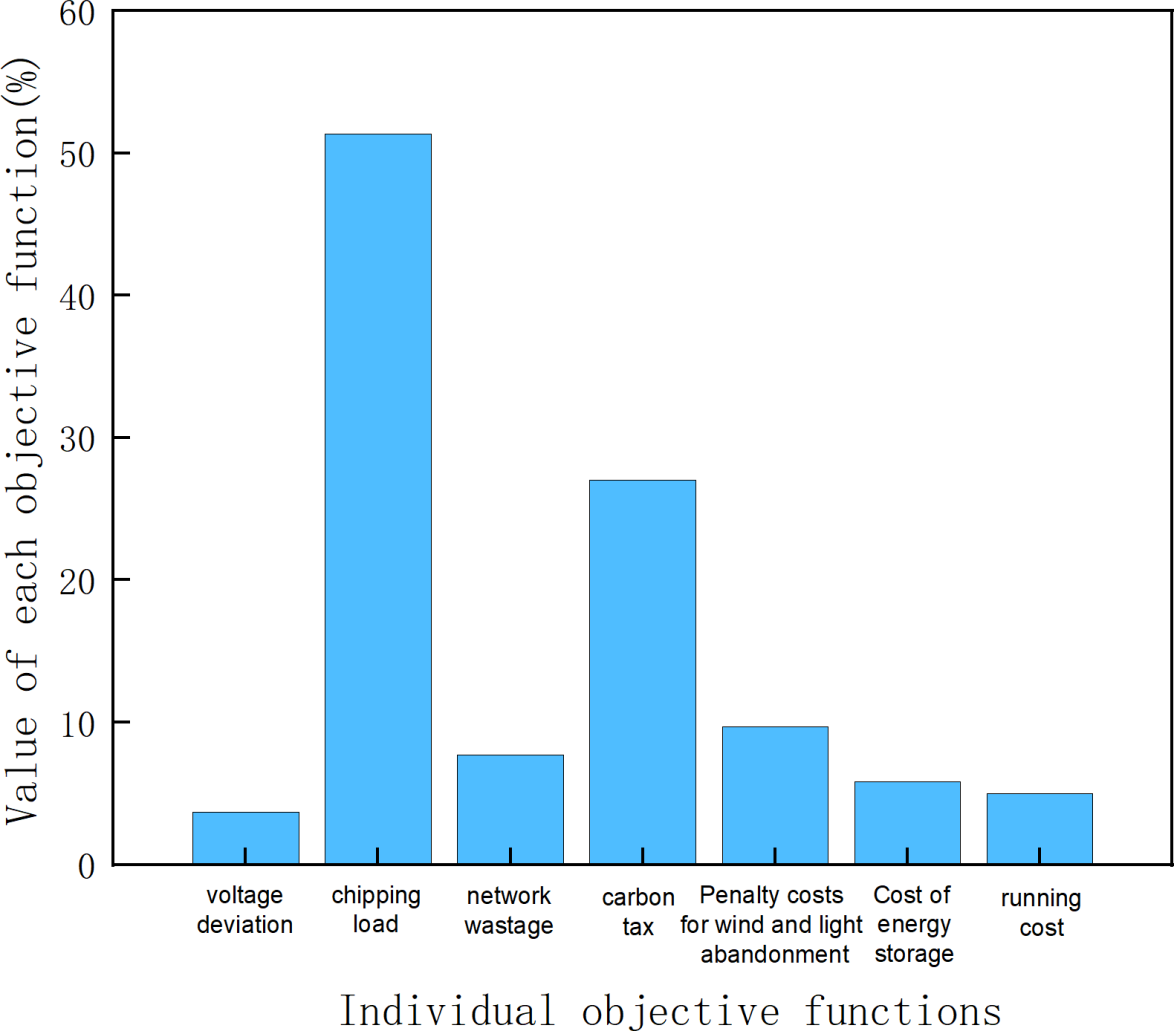
Optimized quantitative indicators for each.

Firstly, the results of the original scheme are evaluated to obtain the indicators of GESS capacity optimization as shown in Fig 8.

In the following, in order to verify the optimal planning of the capacity of gravity energy storage units in the new power system described in this embodiment, a power grid containing wind power, photovoltaic power, thermal power, and gravity energy storage is selected as an arithmetic example, and schemes with different degrees of new energy share are formulated, and the optimal capacity configuration of gravity storage is derived from the optimization by the grasshopper algorithm.Five different data on the capacity share of each power station are shown in Table 1.

**Table 1:** Different energy capacity ratio scenarios.

	Scenario 1	Scenario 2	Scenario 3	Scenario 4	Scenario 5
P_w_	334	385	468	501	550
P_v_	103	151	200	200	350
P_f_	500	450	400	300	200

### 4.3. Capacity analysis of gravity energy storage plant

The optimal installed capacity of gravity energy storage optimized by the grasshopper algorithm for different scenarios and the corresponding minimum cost of the day are shown in Table 2:

**Table 2:** The best installation capacity of pump storage.

Scenario	Percentage of new energy penetration %	Optimal installed capacity	Cost/¥
Scenario 1	43	93	2326490
Scenario 2	50	104	2309879
Scenario 3	57	101	2327860
Scenario 4	66	129	2339020
Scenario 5	72	148	2355350

In Scenario 2, for example, under the new energy penetration ratio of 50% (385 MW for wind farms, 151 MW for PV, and 450 MW configuration for thermal power plants, the cost reaches a minimum of $2,309,879 when the installed capacity of gravity energy storage is 104 MW. When the installed capacity of gravity storage is 106MW, the cost of wind and light abandonment decreases while the cost of energy storage increases, at which point the cost is $23,166,660, which is a small increase compared to the installed capacity of 104WM. When the installed capacity of gravity energy storage is 100WM, the cost of wind and light abandonment penalty increases, and the cost of energy storage decreases, at this time, the total cost is 2307650 yuan, although the cost is cheaper, but due to the volatility of new energy power generation, when it is in the night time can not meet the demand for power supply, so in summary, the new energy rationing under the program, the optimal capacity of gravity energy storage is 104WM. The following figure demonstrates the optimized load and gravity energy storage characteristics.

**Fig 9:**
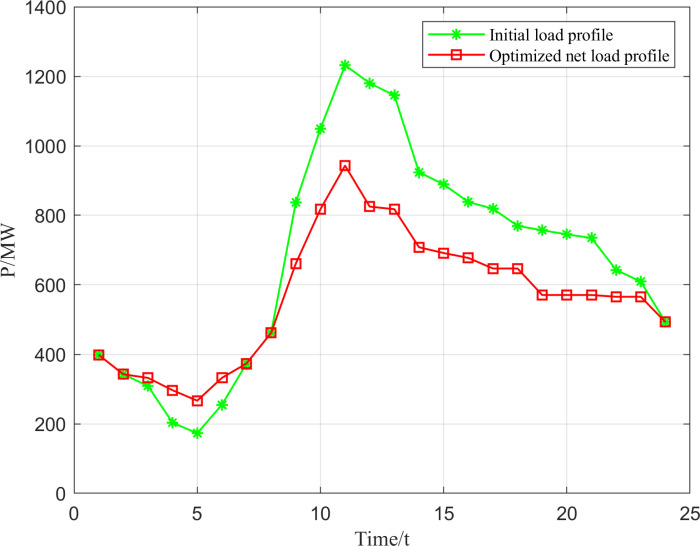
Comparison of network load profiles before and after optimization.

From Fig 9, it can be seen that the maximum peak-to-valley difference in the net load profile of the grid before optimization (when gravity energy storage and optimal abandonment rate are not considered) is 1058.95 MW, while the maximum peak-to-valley difference in the net load after optimization is only 676.365 MW. It can be seen that the gravity energy storage system considering the low-carbon economy can significantly reduce the peak-to-valley difference of the load, successfully realizing the “peak shaving to fill in the valley”, so as to achieve the purpose of reducing the peaking cost of thermal power units.

**Fig 10:**
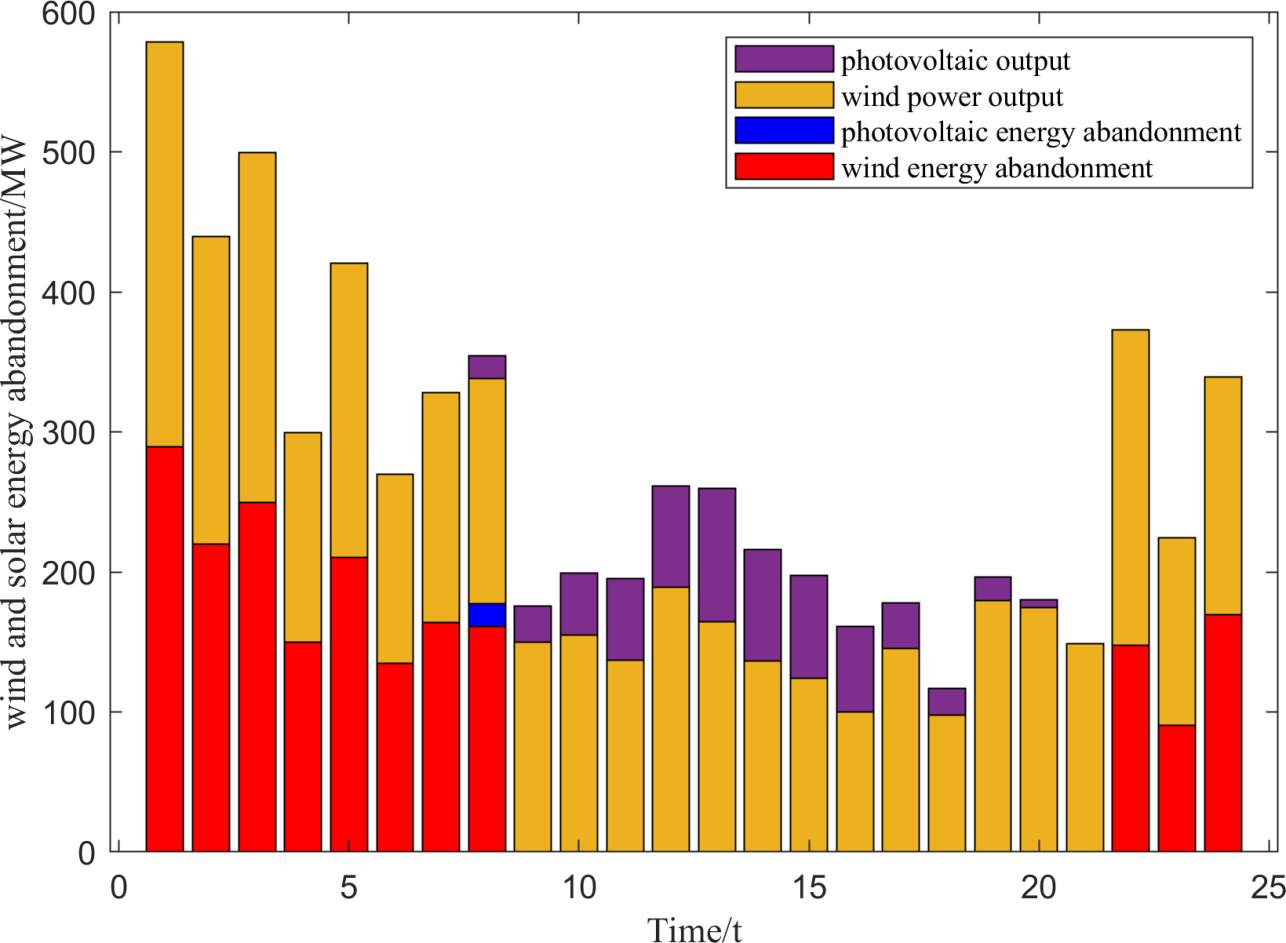
Optimal output of new energy units and wind and solar energy consumption.

**Fig 11:**
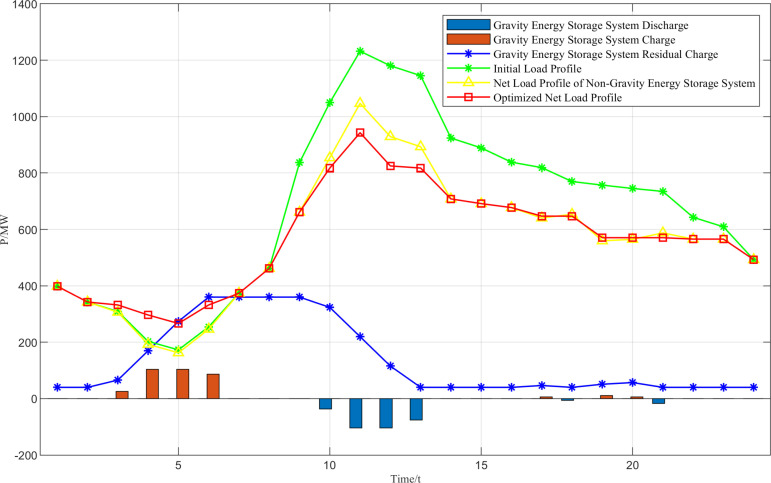
Optimal charging and discharging power of the energy storage plant and the amount of power stored in each time period.

As can be seen in Fig 10, wind abandonment mainly occurs in the time periods 1:00–8:00 and 22:00–24:00, when the optimal wind abandonment rate is maintained at a high level, the maximum abandonment rate has reached 52.83%, while the optimal abandonment rate in other time periods is 0, which is directly related to the anti-peaking characteristics of wind power and the objective function of the upper-level model. This is directly related to the anti-peaking characteristic of wind power and the objective function of the upper model. This is directly related to the anti-peaking characteristic of wind power and the objective function of the upper layer model. Meanwhile, the total amount of abandoned light is small, only 29.46 MW, and the abandoned light time is only 8:00 p.m. The optimal charging and discharging power of the storage plant and the amount of storage power in each time period are shown in Fig 11.

As can be seen from Fig 11, in order to optimize the effect of peak shaving and valley filling, the energy storage station starts charging at 3:00–6:00 and 17:00–20:00 in the low-load period, which improves the “valley value” of the net load curve, and starts discharging in the midday and evening peak hours. Discharging in the afternoon peak and evening peak hours reduces the “peak value” of the net load curve, and its charge-to-power ratio always meets the constraints of energy storage operation, thus easing the peak pressure of thermal power units and increasing the space for wind power and photovoltaic energy consumption.

To validate the robustness and repeatability of the proposed W-GOA method, we conducted 30 independent runs for each scenario with different random seeds. The statistical results, including the mean, standard deviation (SD), and 95% confidence interval (CI) of the total cost, are summarized in Table 3. The results demonstrate that the proposed method consistently achieves low-cost solutions with minimal variability, indicating high reliability.

**Table 3:** Statistical results of total cost (¥) for different scenarios (30 runs).

Scenario	Mean cost	Standard deviation (SD)	95% Confidence interval (CI)
Scenario 1	2,326,490	12,340	[2,324,100; 2,328,880]
Scenario 2	2,309,879	10,890	[2,307,500; 2,312,258]
Scenario 3	2,327,860	11,560	[2,325,300; 2,330,420]
Scenario 4	2,339,020	13,450	[2,336,100; 2,341,940]
Scenario 5	2,355,350	14,780	[2,352,100; 2,358,600]

Furthermore, we compared the proposed W-GOA with the traditional GOA and Adaptive Particle Swarm Optimization (APSO) using a paired t-test. The p-values for all scenarios were less than 0.01, indicating that the performance improvement of W-GOA is statistically significant. Additionally, the coefficient of variation (CV) for W-GOA results across all scenarios was below 0.5%, demonstrating its high repeatability.

## 5. Conclusion

The challenges posed by renewable energy mainly stem from the contradiction between real-time balance of power supply and demand caused by the stochastic nature of wind power, photovoltaic power generation and energy storage output, and the voltage and frequency stabilization problems brought about by grid-connected wind power and photovoltaic. This paper utilizes the advantages of GESS in the transformation of new power structure, compares the cost advantages of conventional PHS and GES in “carbon neutrality and carbon peaking”, and for the first time analyzes the optimal low-carbon and economic installed capacity of the energy storage station from the indicators of the impact of the electric power network, the environmental impact indicators, and the indicators of the economic impact with the use of the multi-objective function.

The main conclusions can be drawn as follows:

(1) By comparing the spiral motion of APSO, GOA and the whale algorithm to the optimized W-GOA to simulate the installed capacity of gravity energy storage under different degrees of new energy penetration in the planning area, it is proved that the convergence and solution speed of the W-GOA are far superior to that of the GOA.
(2) The inefficiency of traditional algorithms in handling nonlinear constraints. The proposed W-GOA algorithm, enhanced by whale spiral motion, demonstrates superior performance in convergence speed and economic outcomes.

Our findings validate GESS as a cost-effective, scalable solution for grids with high renewable penetration, reducing curtailment by 42.3% and carbon emissions by 18%. Future work will extend this framework to multi-regional grids and real-time markets.
